# Extending molecular-replacement solutions with *SHELXE*

**DOI:** 10.1107/S0907444913027534

**Published:** 2013-10-18

**Authors:** Andrea Thorn, George M. Sheldrick

**Affiliations:** a Department of Haematology, University of Cambridge, Cambridge Institute for Medical Research, Wellcome Trust/MRC Building, Hills Road, Cambridge CB2 0XY, England; b Department of Structural Chemistry, Georg-August-Universität Göttingen, Tammannstrasse 4, 37073 Göttingen, Germany

**Keywords:** molecular replacement, density modification, autotracing, *SHELX*

## Abstract

Although the program *SHELXE* was originally intended for the experimental phasing of macromolecules, it can also prove useful for expanding a small protein fragment to an almost complete polyalanine trace of the structure, given a favourable combination of native data resolution (better than about 2.1 Å) and solvent content. A correlation coefficient (CC) of more than 25% between the native structure factors and those calculated from the polyalanine trace appears to be a reliable indicator of success and has already been exploited in a number of pipelines. Here, a more detailed account of this usage of *SHELXE* for molecular-replacement solutions is given.

## Abstract 2

Under favourable circumstances, density modification and polyalanine tracing with *SHELXE* can be used to improve and validate potential solutions from molecular replacement.

## 1. Introduction

The program *SHELXE* (Sheldrick, 2002 ▶, 2008 ▶) was originally designed for the experimental phasing of macromolecules. This required the solution of a marker-atom substructure (heavy atoms or anomalous scatterers) using *F*_A_ values (marker-atom structure-factor amplitudes) estimated from SAD (single-wavelength diffraction), MAD (multi-wavelength anomalous diffraction) or SIRAS (single-wavelength isomorphous and anomalous scattering) data, for example by using the program *SHELXD* (Schneider & Sheldrick, 2002 ▶). The same experimental data also enable phase shifts α to be estimated that can be added to the calculated substructure phases to obtain native phases. In the case of SAD data, these phases are rather approximate and a map calculated from them is not normally interpretable. This is because sufficiently accurate anomalous differences are only available for reflections that do not belong to centrosymmetric projections and often only for the low-resolution data. The phase shifts α can only be estimated as either 90° (if the intensity of reflection *hkl* is greater than that of −*h*−*k*−*l*) or 270° (if the opposite is true). *SHELXE* employs the sphere-of-influence algorithm for density modification to improve these phases (Sheldrick, 2002 ▶). The resulting maps could often be improved further by extrapolating to a higher resolution than had actually been recorded using the free-lunch algorithm (Usón *et al.*, 2007 ▶; Caliandro *et al.*, 2005*a* ▶,*b* ▶). The tracing of a polyalanine backbone, combining the phases from the trace with the experimental phases and repeating the density modification iteratively (Sheldrick, 2010 ▶) often leads to a further substantial improvement in map quality and interpretability. A very useful by-product of this iterative tracing is that the correlation coefficient between the native intensities and those calculated from the trace (‘CC of partial structure against native data’, hereafter abbreviated ‘CC’) proves to be a very reliable guide as to whether the structure has been solved or not. In the case of experimental phasing and native data extending to a resolution of 2.5 Å or better, solutions with a CC of 25% or higher were invariably ‘solved’ (Thorn, 2011 ▶).

This suggests that it might be possible to use the same iterative density modification and backbone tracing to improve phases calculated from a molecular-replacement solution rather than experimental phases, for example in cases where the fragment used is too small or not accurate enough to allow the structure to be completed by the usual refinement procedures. If *SHELXE* is used to expand a large number of trial structures in an automated fashion, the CC > 25% criterion provides a quick way to identify solutions. These may not necessarily correspond to the best starting fragments, but it is the final phases and trace that are useful. In this paper, we explore the use of *SHELXE* to expand small fragments from molecular replacement rather than its more usual applications in experimental phasing, with a view to establishing the limitations of this approach. The pipelines *ARCIMBOLDO* (Rodríguez *et al.*, 2012 ▶) and *AMPLE* (Bibby *et al.*, 2012 ▶) already use *SHELXE* in this way starting from a large number of molecular-replacement solutions for small fragments.

To run *SHELXE*, a reflection file name.hkl in standard *SHELX* format and a partial structure (*e.g.* from molecular replacement) in a PDB-format file name.pda are required. The final phases are written to name.phs, the polyalanine trace to name.pdb and a detailed listing to name.lst. A summary of the progress appears on the console. The program is started with shelxe name.pda followed on the same line by optional switches, *e.g.* -s0.5 to set the solvent content to 0.5 and -a20 for 20 global cycles of density modification and autotracing. There are sensible defaults for most of the options. This simple command-line input makes it particularly easy to call the program as part of an automated pipeline such as *ARCIMBOLDO*, *AMPLE* or *Auto-Rickshaw* (Panjikar *et al.*, 2005 ▶, 2009 ▶). If started without a data filename, the program outputs only a summary of possible command-line options and their defaults to the console.

## 2. Results and discussion

### 2.1. Data normalization and the correlation coefficient CC

For statistical purposes, the observed native structure factors *F*_o_ are normalized to give *E*_o_ such that the mean value of *E*_o_^2^ is unity in all resolution shells. All electron-density maps are calculated with amplitudes *w*(*E*·*F*)^1/2^ and phases ϕ_c_. The native data are corrected for anisotropy using the method of Sheriff & Hendrickson (1987 ▶), which is designed to achieve a mean value of *E*_o_^2^ equal to one for all directions in reciprocal space. The Pearson product–moment correlation coefficient CC between *E*_o_ and the *E*-values *E*_c_ calculated for a partial structure is computed using
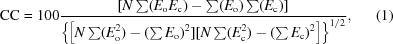
where *N* is the number of reflections [rearranged from the formula used by Fujinaga & Read (1987 ▶) for ease of computation].

### 2.2. Starting-fragment optimization

Two options are provided in *SHELXE* for optimizing an initial fragment from molecular replacement before the first round of density modification. Both are designed to maximize the CC between the observed *E*-values and those calculated for the fragment by a point-atom structure-factor calculation (without the use of atomic displacement parameters *B*). One advantage of using *E*-values is that they should correspond theoretically to a point-atom structure.

The command-line switch -O performs a rigid-group refinement to optimize the CC. In this work, the CC values are always calculated between *E*_o_ and *E*_c_ by the method of Fujinaga & Read (1987 ▶). The input fragment may be divided up into several rigid groups by means of ‘REMARK DOMAIN *N*’ instructions (where *N* is the domain number) in the input PDB-format file name.pda. The rigid groups do not need to be contiguous in this file. A random-start simplex algorithm is used to search for the best fit for fragments in the region of their starting positions. This works best when the rigid groups are whole domains or α-helices, but it is not recommended for individual β-strands.

The command-line switch -o instructs the program to eliminate up to a certain number of amino-acid residues to obtain the largest increase in the CC. This is repeated iteratively until no further improvement can be achieved. This option can be useful when the fragment used for molecular replacement is only partially correct. If either or both of these options are employed, the optimized starting model is written to the PDB-format file name.pdo. After optional optimization of the starting fragment, a point-atom structure-factor summation is performed to generate the starting phases. Each phase is assigned an initial weight using the σ_A_ formula (Read, 1986 ▶; Sheldrick, 2002 ▶).

### 2.3. Density modification with the sphere-of-influence algorithm

The idea of density modification is to modify the electron density so that it looks more like what is to be expected for a typical macromolecule. Fourier inversion of the modified density should then result in improved phases. Density modification will be more effective when the density comes from independent (albeit noisy) experimental phases than when it is based on phases calculated from a fragment, *e.g.* a molecular-replacement solution. In the latter case the density could look like a macromolecule, even if it has the wrong position in the unit cell; the translation function in molecular replacement can have several potential solutions. Most density-modification procedures begin by dividing the map into solvent and protein (or other macromolecular) regions and apply different modifications to the density in the two regions. The sphere-of-influence algorithm in *SHELXE* (Sheldrick, 2002 ▶) is more flexible, but works best for relatively high-resolution data (say 2.5 Å or better).

The sphere of influence is a spherical shell of radius 2.42 Å about a voxel (a grid point) in the density map. If there is a large variance in the density values in this spherical shell, the voxel is more likely to correspond to an atomic position in the macromolecule, because 2.42 Å is a common 1,3-interatomic distance in proteins, polynucleotides, sugars *etc.* For such voxels, negative density is truncated to zero, but positive density is left unchanged. If the variance of the density at the spherical surface is low, the voxel is more likely to be in the solvent region, in which case the density is ‘flipped’ (*i.e.* it is changed from positive to negative or *vice versa* without changing its absolute magnitude). After several cycles, this results in flattening of the solvent regions. Solvent flipping (Abrahams, 1997 ▶) is widely used in density-modification programs to combat model bias. For intermediate values of the variance, a weighted mean of these two operations is performed. This results in a ‘fuzzy’ solvent boundary that is less likely than a sharp boundary to lock the program into a false solution.

Each global cycle consists of density modification followed by autotracing. By default 20 cycles of density modification are employed in each global cycle. This is usually a good choice, but it might be worth increasing if the solvent content is very high.

### 2.4. Generating a polyalanine trace

Before starting the tracing, a no-go map is constructed. This initially contains just tubes along any pure rotation axis that passes through the unit cell, but later also contains the regions already successfully traced. It also temporarily contains the current trace, to avoid the possibility of a never-ending trace around a closed path. In the case of experimental phasing it also contains the heavy-atom positions. This map enables false tracing steps to be avoided efficiently.

Each trace starts from a tripeptide or (if -q was specified) a seven-residue α-helix fragment that has been placed to give a good fit to the density and to avoid the no-go regions. The number of random search positions tried can be increased with, for example, -t10 (which would take ten times longer). However, this is the least efficient step in the program and needs further optimization (but fortunately it would be easy to perform it in parallel). The trace is performed by a simplex search with a two-residue look-ahead, varying the backbone torsion angles starting from angles common in a Ramachandran diagram and also allowing the N—C^α^—C τ angles to vary over a limited range (Touw & Vriend, 2010 ▶). When the trace cannot be extended further at either end, an empirical figure of merit is calculated. This takes into account the length of the trace, the overall fit to the density, the presence of density about 2.9 Å from the backbone N atoms in the N—H direction (representing N—H⋯O hydrogen bonds), the presence of a well defined secondary structure and the overall Ramachandran fit. Only traces with good figures of merit are retained and added to the no-go map.

Finally, the surviving traces are spliced where appropriate to bridge small gaps and to avoid close contacts. Further details may be found in Sheldrick (2010 ▶). After completing the trace, one *B* value (isotropic displacement parameter) is refined per residue and the CC against the native data is calculated. σ_A_ weights (Read, 1986 ▶) are used to combine the resulting phases with those from the density modification and the next global cycle is started.

### 2.5. Summary of workflow

*SHELXE* always uses σ_A_ weights based on the CC between the calculated and native *E*-values as a function of resolution to combine different sources of phase information (see Sheldrick, 2002 ▶ and §[Sec sec2.1]2.1). The overall workflow is as follows.

(i) The native intensities are merged to produce a unique anisotropically scaled list of native structure factors *F*_T_ with the systematic absences removed. Corresponding normalized structure factors *E*_T_ are calculated.
(ii) Starting fragments are optionally optimized as explained in §[Sec sec2.2]2.2, followed by calculation of their phase contribution ϕ_frag_ and σ_A_ weights *w*_frag_.
(iii) If anomalous differences are provided, the anomalously scattering atoms are located as described by Thorn & Sheldrick (2011 ▶) and used to estimate a phase contribution ϕ_SAD_ with σ_A_ weights *w*_SAD_.
(iv) If ϕ_SAD_ is available, ϕ_SAD_ and *w*_SAD_ are combined with ϕ_frag_ and *w*_frag_ to give starting phases ϕ_comb_ and *w*_comb_,
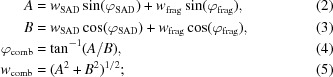
if the resulting *w*_comb_ is greater than 1.0 then it is replaced by 1.0.
(v) Density modification and auto-tracing.
  (1) Starting from ϕ_comb_ and *w*_comb_, *m* (typically 20) cycles of density modification according to the sphere-of-influence algorithm (Sheldrick, 2002 ▶) are performed, resulting in ϕ_dm_ and w_dm_.
  (2) If the specified number of cycles of auto-tracing has already been performed, exit.
  (3) Otherwise, a no-go map is generated to avoid tracing through the anomalous scatterers (if any) or too close to symmetry elements. As the tracing progresses, this no-go map is modified to avoid repeatedly tracing the same region or generating a cyclic polypeptide.
  (4) A map based on phases ϕ_dm_ and amplitudes *w*_dm_(*F*_T_·*E*_T_)^1/2^ is calculated and traced as described by Sheldrick (2010 ▶), taking the no-go map into account.
  (5) Refinement of the isotropic displacement parameters *B* (one per amino acid or SAD atom) for the trace and calculation of the CC for the *E*-values calculated from the traced fragments (including SAD atoms, if any) against *E*_T_. When this CC exceeds 25%, the structure is almost certainly solved (for data to 2.5 Å resolution or better). The traced fragments are used to calculate new ϕ_frag_ and *w*_frag_ values.
  (6) If the CC value is the best so far, output the polyalanine trace as well as ϕ_dm_ and *w*_dm_.
  (7) The phases from the trace are combined with those from density modification using the same procedure as in step (iv), but *w*_dm_ is multiplied by a factor that may be specified on the command line (the default is 0.8). The resulting phases are then combined with the SAD phases (if any) in the same way. Then go to step (1) of step (v).

### 2.6. Selected examples

A number of typical correct and incorrect molecular-replacement solutions were generated using the program *Phaser* (McCoy *et al.*, 2007 ▶) with data for concanavalin A (in-house data similar to PDB entry 2g4i; Mueller-Dieckmann *et al.*, 2007 ▶), which was chosen because it consists predominantly of β-strands, which are known to be more difficult than α-­helices for tracing algorithms.

**Figure 1:**
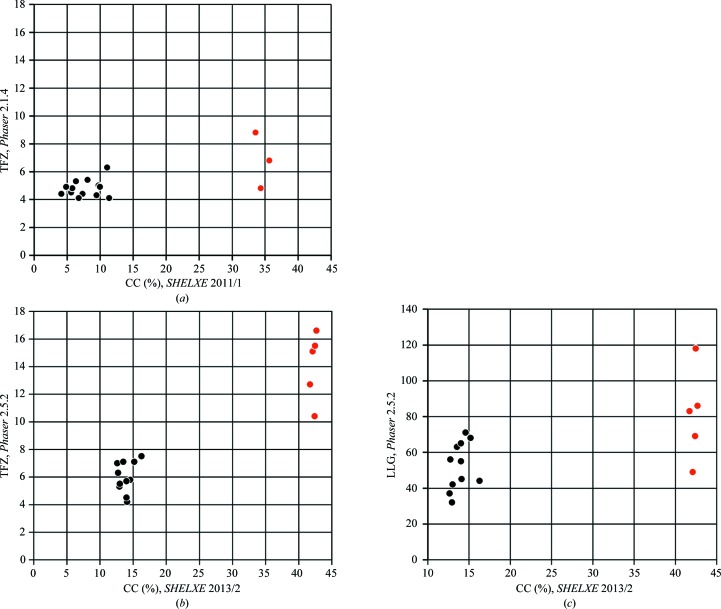
Figures of merit for the same set of starting models using different versions of *Phaser* and *SHELXE*: (*a*) TFZ against CC for *Phaser* 2.1.4 and *SHELXE* 2011/1 as in Thorn (2011 ▶), (*b*) TFZ against CC for *Phaser* 2.5.2 and *SHELXE* 2013/2 and (*c*) LLG against CC for *Phaser* 2.5.2 and *SHELXE* 2013/2. The CC values clearly identify the correct traces (on the right-hand side) after *SHELXE*, but the TFZ values from the new *Phaser* version 2.5.2 also clearly identify the MR solutions that can be expanded to the full structure.

Fig. 1 ▶ compares the figures of merit TFZ (translation-function *Z*-score) and LLG (log-likelihood gain) from *Phaser* with the final CC value from *SHELXE*. With the most recent versions of both programs the TFZ figure of merit from *Phaser* (version 2.5.2) correctly identifies all of the solutions that *SHELXE* was able to expand to the full correct structure. *SHELXE* 2013/2 was able to successfully expand more starting models and also gave more complete structures with higher CC values than the previous beta test version 2011/1.

**Figure 2:**
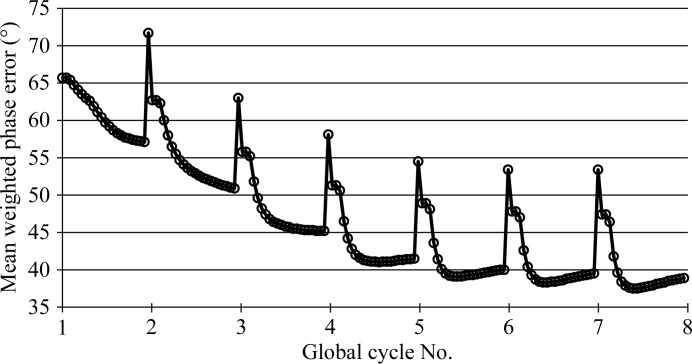
Progress of the mean weighted phase error against the final refined structure for the first six global cycles of one of the successful concanavalin A runs. The sharp peaks correspond to the start of the density modification using new phases obtained by combining the existing phases with those from a new trace.

The progress of the mean weighted phase error in terms of the global cycle number is illustrated for one of the con­canavalin A runs in Fig. 2 ▶. There is a sharp peak once in each global cycle when phases from the new polyalanine trace are combined with the existing phases, but then a rapid drop in the phase error when the combined phases are subjected to density modification. It can be seen that both the density modification and the tracing contribute to the overall reduction in the phase error, and that in this particular case it might have been better to perform more density-modification iterations in the initial global cycles and fewer in the later stages.

**Figure 3:**
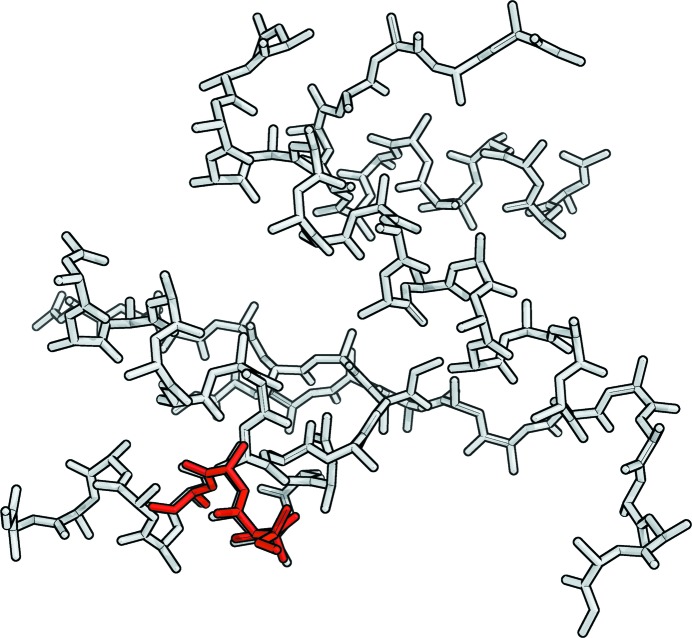
A four-amino-acid starting fragment (red) and the final trace (grey) for PDB entry 1zzk (solvent content 33.4%; space group *C*2; resolution 0.95 Å). This illustrates the ability of the program to expand from a very small but accurately placed molecular-replacement fragment to essentially the full structure, given favourable conditions. This figure was generated with *PyMOL* (DeLano, 2002 ▶).

The ability of the method to expand from a very small but rather accurately placed fragment is illustrated in Fig. 3 ▶, in contrast to concanavalin where the fragments leading to structure solution account for large portions of the final structure. The MR solution (in red) consisted of only four amino acids and the final trace from *SHELXE* (in grey) contained 86 amino acids, four more than in the deposited structure (PDB entry 1zzk; Backe *et al.*, 2005 ▶).

**Figure 4:**
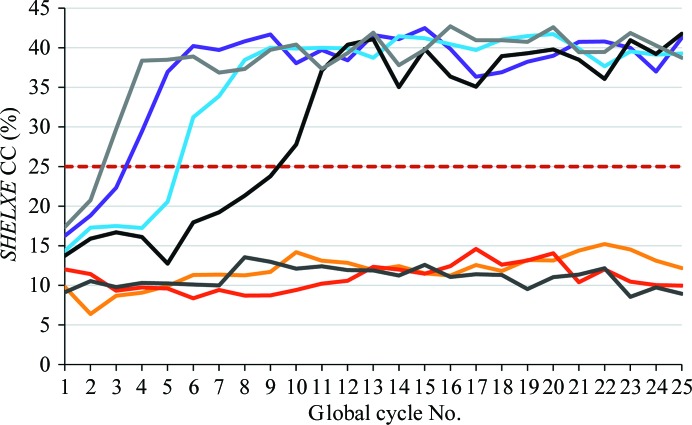
Evolution of the CC as a function of the global cycle number starting from a selection of MR models (19–116 residues) for concanavalin A (237 residues). The dashed line at 25% shows the threshold that gives a reliable indication as to whether the structure has been solved. Here, the MR models yielding successful *SHELXE* solutions were pruned core folds of structures of sequential similarity: garden pea lectin (PDB entry 2ltn; Prasthofer *et al.*, 1989 ▶) and arcelin-5 (PDB entry 1ioa; Hamelryck *et al.*, 1996 ▶), 94–116 residues.

The development of the CC with global cycle number is illustrated in Fig. 4 ▶ for several of the concanavalin A runs. For the successful runs, there is a relatively sharp and substantial increase in CC over the course of a small number of cycles, but different numbers of global cycles are needed to reach this point. In other examples we have found that 20 or more global cycles were needed to reach this point. There is a very clear distinction between solutions and non-solutions separated by the threshold CC value of 25% (dashed line) that appears to be a reliable general criterion for success. In this particular case, the initial CC values for the starting fragments that led to successful solutions were generally higher than those that could not be expanded, showing that the CC value is able to give some indication of which models are at least partially correct even when the mean weighted phase errors are greater than 70°.

### 2.7. MRSAD

If anomalous differences have been measured but were too weak for SAD phasing, the heavy-atom positions can often still be located by reversing the procedure used for SAD phasing. Instead of adding the 90 or 270° phase shift α to the heavy-atom phases to obtain approximate native phases, α can be subtracted from the native phases to obtain approximate heavy-atom phases. Experience with the program *ANODE* (Thorn & Sheldrick, 2011 ▶) suggests that even a very weak anomalous signal can give significant heavy-atom density. *SHELXE* has been adapted to find the heavy atoms from a partial molecular-replacement solution and to then use them to improve the native phases. However, there have not yet been any reports of this addition to *SHELXE* being successful in solving a previously unknown structure.

## 3. Conclusions

*SHELXE* is used in several structure-solution pipelines as a means of quickly and efficiently identifying correct molecular-replacement solutions, reducing model bias and expanding the structure.

Both data resolution and the solvent content have a significant influence on the chances of success of both the density modification and the polyalanine tracing. Although *SHELXE* can still trace some structures with data to 3 Å resolution and a high solvent content given high-quality MAD data, structure expansion from a small fragment without any experimental phase information requires data of at least 2.1 Å resolution. Despite the use of anisotropic scaling, it seems that *SHELXE* is, in general, less effective in triclinic and monoclinic space groups, as well as for purely β-sheet structures.

While for borderline cases there is an element of chance as to whether the tracing will succeed or not and there have been appreciable differences in performance between different versions of *SHELXE*, a CC of partial structure against native data of greater than 25%, given native data of 2.5 Å resolution or better, invariably indicates a successful structure solution.

## 4. Availability

*SHELX* is available free of charge for academic use from http://shelx.uni-ac.gwdg.de/SHELX/, where extensive documentation may also be found.

## References

[bb1] Abrahams, J. P. (1997). *Acta Cryst.* D**53**, 371–376. 10.1107/S0907444996015272 15299902

[bb21] Backe, P. H., Messias, A. C., Ravelli, R. B. G., Sattler, M. & Cusack, S. (2005). *Structure*, **13**, 1055–1067. 10.1016/j.str.2005.04.008 16004877

[bb3] Bibby, J., Keegan, R. M., Mayans, O., Winn, M. D. & Rigden, D. J. (2012). *Acta Cryst.* D**68**, 1622–1631. 10.1107/S0907444912039194 23151627

[bb4] Caliandro, R., Carrozzini, B., Cascarano, G. L., De Caro, L., Giacovazzo, C. & Siliqi, D. (2005*a*). *Acta Cryst.* D**61**, 556–565. 10.1107/S090744490500404X 15858265

[bb20] Caliandro, R., Carrozzini, B., Cascarano, G. L., De Caro, L., Giacovazzo, C. & Siliqi, D. (2005*b*). *Acta Cryst.* D**61**, 1080–1087. 10.1107/S0907444905015519 16041073

[bb5] DeLano, W. L. (2002). *PyMOL* http://www.pymol.org.

[bb6] Fujinaga, M. & Read, R. J. (1987). *J. Appl. Cryst.* **20**, 517–521.

[bb26] Hamelryck, T., Poortmans, F., Goossens, A., Angenon, G., Van Montagu, M., Wyns, L. & Loris, R. (1996). *J. Biol. Chem.* **271**, 32796–32802. 10.1074/jbc.271.51.32796 8955116

[bb30] McCoy, A. J., Grosse-Kunstleve, R. W., Adams, P. D., Winn, M. D., Storoni, L. C. & Read, R. J. (2007). *J. Appl. Cryst.* **40**, 658–674. 10.1107/S0021889807021206 PMC2483472 19461840

[bb22] Mueller-Dieckmann, C., Panjikar, S., Schmidt, A., Mueller, S., Kuper, J., Geerlof, A., Wilmanns, M., Singh, R. K., Tucker, P. A. & Weiss, M. S. (2007). *Acta Cryst.* D**63**, 366–380. 10.1107/S0907444906055624 17327674

[bb7] Panjikar, S., Parthasarathy, V., Lamzin, V. S., Weiss, M. S. & Tucker, P. A. (2005). *Acta Cryst.* D**61**, 449–457. 10.1107/S0907444905001307 15805600

[bb8] Panjikar, S., Parthasarathy, V., Lamzin, V. S., Weiss, M. S. & Tucker, P. A. (2009). *Acta Cryst.* D**65**, 1089–1097. 10.1107/S0907444909029643 PMC2756167 19770506

[bb25] Prasthofer, T., Phillips, S. R., Suddath, F. L. & Engler, J. A. (1989). *J. Biol. Chem.* **264**, 6793–6796. 2708344

[bb9] Read, R. J. (1986). *Acta Cryst.* A**42**, 140–149.

[bb10] Rodríguez, D., Sammito, M., Meindl, K., de Ilarduya, I. M., Potratz, M., Sheldrick, G. M. & Usón, I. (2012). *Acta Cryst.* D**68**, 336–343. 10.1107/S0907444911056071 PMC3322593 22505254

[bb11] Schneider, T. R. & Sheldrick, G. M. (2002). *Acta Cryst.* D**58**, 1772–1779. 10.1107/s0907444902011678 12351820

[bb12] Sheldrick, G. M. (2002). *Z. Kristallogr.* **217**, 644–650.

[bb13] Sheldrick, G. M. (2008). *Acta Cryst.* A**64**, 112–122. 10.1107/S0108767307043930 18156677

[bb14] Sheldrick, G. M. (2010). *Acta Cryst.* D**66**, 479–485. 10.1107/S0907444909038360 PMC2852312 20383001

[bb15] Sheriff, S. & Hendrickson, W. A. (1987). *Acta Cryst.* A**43**, 118–121.

[bb16] Thorn, A. (2011). PhD thesis. Georg-August-Universität Göttingen, Germany. http://hdl.handle.net/11858/00-1735-0000-0006-B072-8.

[bb17] Thorn, A. & Sheldrick, G. M. (2011). *J. Appl. Cryst.* **44**, 1285–1287. 10.1107/S0021889811041768 PMC3246834 22477786

[bb18] Touw, W. G. & Vriend, G. (2010). *Acta Cryst.* D**66**, 1341–1350. 10.1107/S0907444910040928 PMC2995724 21123875

[bb19] Usón, I., Stevenson, C. E. M., Lawson, D. M. & Sheldrick, G. M. (2007). *Acta Cryst.* D**63**, 1069–1074. 10.1107/S0907444907042230 17881824

